# Imaging Parameters for Contralateral Hip Dysplasia in Asymptomatic Adults Over 60 Years Old With Femoral Neck Fractures

**DOI:** 10.1111/os.70203

**Published:** 2025-11-06

**Authors:** Zhiqiang Chen, Zhendong Zhang, Hui Cheng, Ziyin Xu, Wei Chai, Dianzhong Luo, Hong Zhang

**Affiliations:** ^1^ School of Medicine Nankai University Tianjin China; ^2^ Senior Department of Orthopaedics The Fourth Medical Center of PLA General Hospital Beijing China

**Keywords:** developmental dysplasia of the hip, femoral neck fracture, older adults, radiographic analysis

## Abstract

**Objective:**

It is unclear whether a hip with a developmental dysplasia deformity can remain functional and free of osteoarthritis (OA) throughout life. This study aims to determine the percentage of Chinese older adults without severe OA who meet the diagnostic criteria for DDH and BDDH. Additionally, this study seeks to explore hip morphology in this population.

**Methods:**

Data from 808 consecutive patients with recent unilateral femoral neck fractures, collected between January 2022 and October 2024, were retrospectively analyzed. A total of 493 patients (493 hips) were included in the analysis. For imaging evaluation, the following parameters of the contralateral (unfractured) hip were measured: LCEA, Tönnis angle, Sharp's angle, femoral head eminence index (FHEI), and femoral head lateralization. Categorical variables were expressed as numbers and percentages. Continuous variables were presented as mean ± SD if normally distributed; otherwise, they were reported as median (Q1, Q3). The Pearson chi‐square test, likelihood ratio chi‐square test, or Fisher's exact test was used to compare categorical variables. An independent‐samples *t*‐test or Mann–Whitney *U* test was used to compare continuous variables in the group analysis. For parameter comparisons between multiple groups, use ANOVA with post hoc analysis.

**Results:**

In this study, 7.1% of individuals aged over 60 years with unilateral femoral neck fractures exhibited imaging results consistent with DDH, while 13.2% had results consistent with BDDH. Patients with DDH and BDDH were at a higher risk of developing mild OA compared to individuals with normal acetabular coverage. However, not all individuals with DDH or BDDH develop OA. Only the Tönnis angle was significantly associated with mild OA, indicating a 7.8% increase in OA risk for each 1° increase in the Tönnis angle. Significant differences were observed in the Tönnis angle (5.0 ± 3.9 vs. 11.5 ± 4.1 vs. 17.5 ± 4.1, *p* < 0.001), Sharp's angle (38.2 ± 2.9 vs. 41.7 ± 2.5 vs. 44.0 ± 2.5, *p* < 0.001), femoral head lateralization (7.8 ± 2.7 vs. 9.3 ± 2.6 vs. 10.3 ± 2.8, *p* < 0.001), and FHEI (17 ± 4 vs. 25 ± 3 vs. 30 ± 4, *p* < 0.001) between the normal group and both the DDH and BDDH groups.

**Conclusion:**

The prevalence of DDH imaging abnormalities is notable among Chinese older adults without severe OA. Individuals with DDH and BDDH are more likely to exhibit mild OA symptoms, although not all develop OA. Using multiple imaging parameters in addition to LCEA facilitates characterizing hip morphology in asymptomatic individuals with DDH.

## Graphical abstract

The prevalence of DDH imaging abnormalities is not low among Chinese older adults with healthy hip function. Individuals with DDH and BDDH are more likely to present with mild OA symptoms, but not all individuals develop OA.

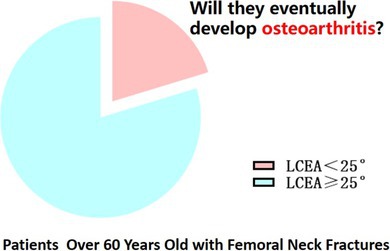

## 1 Introduction

Developmental dysplasia of the hip (DDH) is a deformity in which the acetabulum does not adequately cover the femoral head, leading to increased joint contact stresses and abnormal loading patterns. These conditions predispose the hip to osteoarthritis (OA) [1, 2, 3]. DDH diagnosis is based on the lateral center‐edge angle (LCEA) measured on anteroposterior (AP) pelvic radiographs [4]. An LCEA < 20° is diagnostic of DDH, whereas an LCEA between 20° and 25° is categorized as borderline DDH (BDDH) [5, 6, 7, 8]. The standard surgical treatments for DDH or BDDH include periacetabular osteotomy (PAO) and hip arthroscopy (HAS), with the aim of improving hip coverage and maintaining joint stability to delay the development of OA [9, 10, 11].

DDH occurs not only in symptomatic patients but also in asymptomatic individuals without severe OA. The prevalence of DDH diagnosed through imaging data in the general population ranges from 3% to 15% [1, 12, 13, 14]. Contrarily, the prevalence of BDDH ranges from 17% to 25% [1, 12, 15, 16]. These studies enrolled patients across wide age ranges, that is, 22–93 years [1], 18–50 years [13], 20–70 years [14], 19 years [12], and > 45 years [15]. Interestingly, most studies have focused on mixed‐age or young populations, with relatively few studies elucidating the prevalence of DDH in older adults. If older adults with DDH remain asymptomatic after decades of work and daily life, it is more certain that their deformity will not lead to severe OA. In clinical practice, several older adults without severe OA meeting the diagnostic criteria for DDH imaging have been identified. However, the proportion of Chineseolder adults without severe OA meeting the DDH imaging diagnostic criteria remains unclear. Additionally, the morphological abnormalities of the hip joints in Chinese older adults—after decades of regular work without progression to symptomatic hip OA—remain elusive.

To further investigate the morphology of the hip joints in older adults, AP pelvic radiographs from older adults with unilateral femoral neck fractures were analyzed. Furthermore, the imaging morphology of the fractured contralateral hip was assessed to determine the percentage of Chinese older adults with Tönnis OA grade 0/1 meeting the diagnostic criteria for DDH and BDDH. We believe they are asymptomatic individuals without severe osteoarthritis, and their hip function is healthy. If DDH occurs at a high rate in asymptomatic older adults, it may refute the screening of asymptomatic populations for DDH and premature interventional surgical treatment.

Additionally, this study seeks to explore the distribution patterns of DDH‐related imaging parameters on AP pelvic radiographs in this population.

## 2 Patients and Methods

### 2.1 Study Design and Setting

This was a retrospective study performed at the Fourth Medical Center of PLA General Hospital in China (a national tertiary referral center).

### 2.2 Patients

**FIGURE 1:**
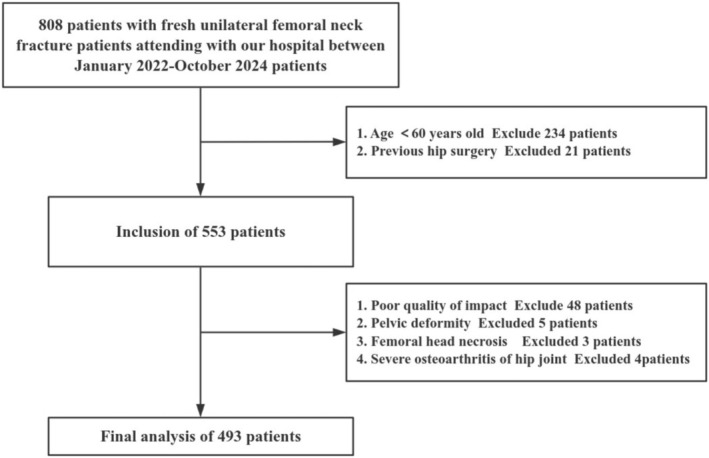
This flow chart depicts the selection of the study population.

Data from 808 consecutive patients with recent unilateral femoral neck fractures from January 2022 to October 2024 were retrospectively collected. The inclusion criteria were as follows: (i) age ≥ 60 years and (ii) no prior hip surgery. The exclusion criteria were as follows: (i) poor image quality (e.g., pelvic rotation, tilt, and blurring that could not be measured accurately, see section “Radiographic measurements”), (ii) pelvic deformity, (iii) femoral head necrosis, and (iv) OA of the contralateral hip with a Tönnis grade > 1. Finally, 493 patients (493 hips) were analyzed (Figure 1). The mean age was 77.1 years (range: 60–98 years), with 361 women (73%). A total of 268 patients (54%) had left‐sided femoral neck fractures, and the mean body mass index (BMI) was 23.0 ± 3.8 kg/m^2^.

### 2.3 Radiographic Measurements

**FIGURE 2:**
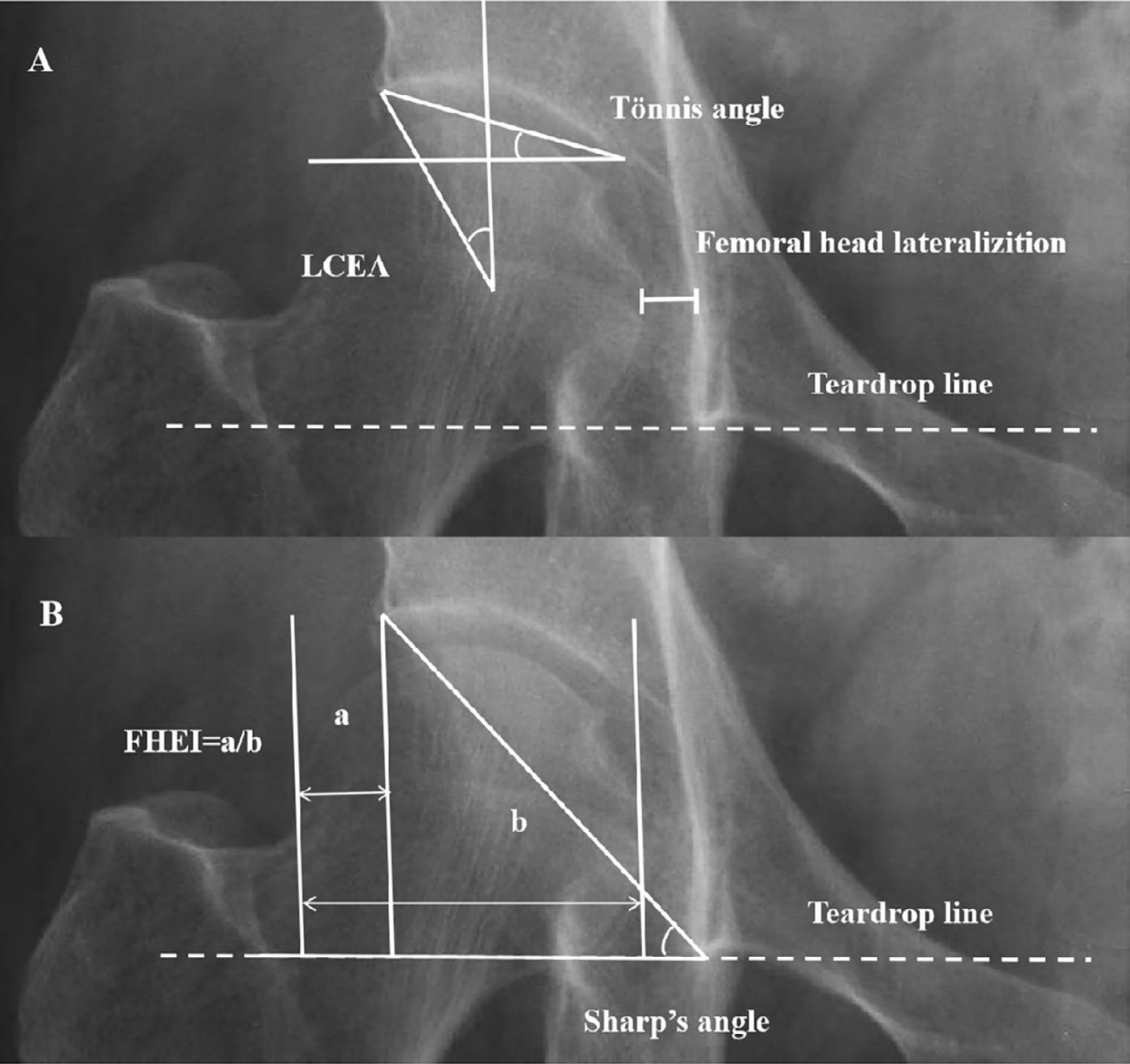
Measurement of each imaging index. (A) LCEA: The angle between a plumb line passing through the center of the femoral head and connecting the center and the upper outer edge of the acetabulum. Tönnis angle: The angle between a line passing through the inner and outer edges of the brow arch and a horizontal line parallel to the transverse axis of the pelvis. Femoral head lateralization: The shortest distance between the innermost edge of the femoral head and the iliac sitting line. (B) Sharp's angle: The angle between a line connecting the distal end of the acetabular teardrop and the outermost point of the acetabulum and the pelvic horizontal line. FHEI: The ratio of the transverse diameter of the femoral head extending beyond the acetabulum to the femoral head diameter.

AP pelvic radiographs were analyzed with patients placed supine. The X‐ray tube‐to‐film distance was set at 120 cm, with the tube oriented perpendicular to the table. The crosshairs of the beam were centered at a point midway between the superior border of the pubic symphysis and a line connecting the anterior superior iliac spines. For imaging evaluation, the LCEA, Tönnis angle, Sharp's angle, femoral head eminence index (FHEI), and femoral head lateralization of the contralateral (unfractured) hip were measured (Figure 2). LCEA is defined as the angle between a plumb line passing through the center of the femoral head and connecting the center and the upper outer edge of the acetabulum [7]. Since excessive pelvic rotation may affect LCEA measurements [17], the obturator index (FOI) is used to determine the degree of pelvic rotation in the axial plane. The FOI is calculated as the ratio of the widest horizontal diameter of the right obturator foramen to the widest horizontal diameter of the left obturator foramen [18]. Only images with FOI values between 0.7 and 1.7 are retained to ensure the accuracy of LCEA measurement results. The Tönnis angle is defined as the angle between a line passing through the inner and outer edges of the brow arch and a horizontal line parallel to the transverse axis of the pelvis [18]. Sharp's angle is defined as the angle between a line connecting the distal end of the acetabular teardrop and the outermost point of the acetabulum and the pelvic horizontal line [19]. FHEI is calculated as the ratio of the transverse diameter of the femoral head extending beyond the acetabulum to the femoral head diameter [20]. Femoral head lateralization is defined as the shortest distance between the innermost edge of the femoral head and the iliac sitting line [21]. For easy interpretation, patients were categorized into four groups: the DDH group (LCEA < 20°), the BDDH group (20° ≤ LCEA < 25°), the normal acetabular coverage group (25° < LCEA ≤ 40°, abbreviated as the normal group) and the acetabular overcoverage group (LCEA > 40°). This study utilized imaging only for categorizing and not for clinical diagnosis. The Tönnis OA grade was used to assess hip OA severity. Two orthopedic surgeons (author 1 and author 2) independently measured all data to assess inter‐observer agreement. Both doctors received training to ensure consistency in measurement standards. Author1 repeated the measurements after 1 month to assess intra‐observer agreement. All ICC values exceed 0.9, with the specific data presented in Table 5.

### 2.4 Ethical Approval

Ethical approval for this study was obtained from the Ethics Review Board (number 2023KY002‐KS001) in the Department of Orthopedics, the Fourth Medical Center of PLA General Hospital.

### 2.5 Statistical Analysis

Before data collection, a power analysis showed that 450 subjects were needed to obtain a dysplasia prevalence with a precision of ±3% unit. Agreement for continuous variables was assessed using the intraclass correlation coefficient, with values < 0.5 indicating poor agreement, 0.5–0.75 indicating moderate agreement, 0.75–0.9 indicating good agreement, and > 0.9 indicating excellent agreement. Categorical variables were expressed as numbers and percentages. Continuous variables were presented as mean ± SD if normally distributed; otherwise, they were reported as median (Q1, Q3). The Pearson chi‐square test, likelihood ratio chi‐square test, or Fisher's exact test was used to compare categorical variables. An independent‐samples *t*‐test or Mann–Whitney *U* test was used to compare continuous variables in the group analysis. For parameter comparisons between multiple groups, use ANOVA with post hoc analysis. Statistical analysis was performed using SPSS Statistics 29.0.0 (IBM), with *p* value < 0.05 considered statistically significant.

## 3 Results

**FIGURE 3:**
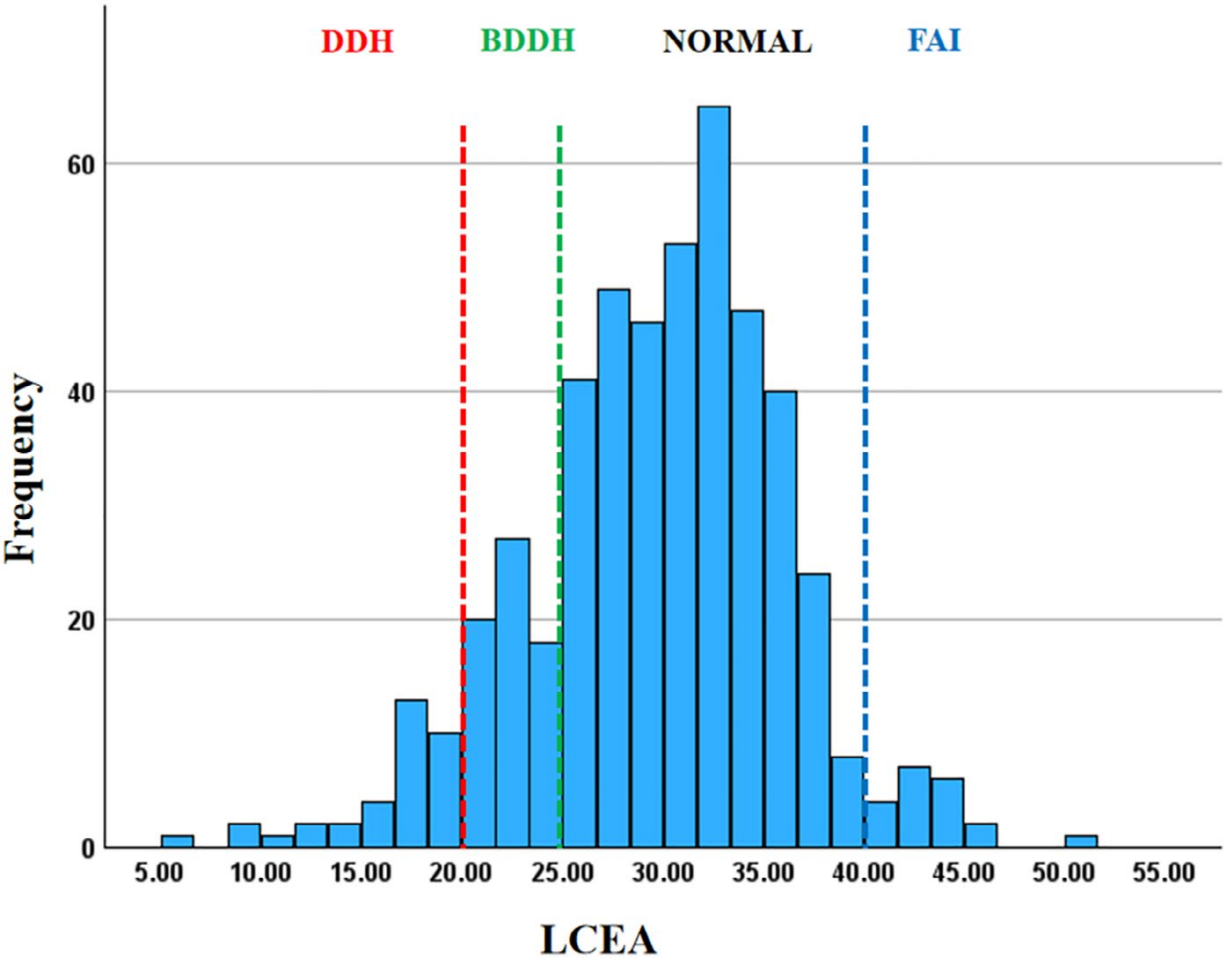
A histogram showing the distribution of all patients by LCEA.

Table 1 summarizes the imaging parameters and BMI of 493 patients. Of them, 100 patients (20.2%) had an LCEA < 25°, including 35 with DDH (7.1%) and 65 with BDDH (13.2%). No difference was observed in the incidence of DDH and BDDH between men and women (7.6% vs. 6.9%, *p* = 0.80; 13.6% vs. 13.0%, *p* = 0.86). Furthermore, 20 patients (4.1%) had an LCEA > 40°. Figure 3 illustrates LCEA distribution patterns in all patients.

**TABLE 1:** Imaging measurements of all patients.

Group	LCEA in °	Tönnis angle in °	Sharp's angle in °	Femoral head lateralization in mm	FHEI in %	Tönnis Grade 1	BMI in kg/m^2^
DDH (*n* = 35)	17.3 (15.3, 18.4)	17.5 ± 4.1	44.0 ± 2.5	10.3 ± 2.8	30 ± 4	37.1 (13)	22.9 ± 3.9
BDDH (*n* = 65)	22.6 (21.3, 23.6)	11.5 ± 4.1	41.7 ± 2.5	9.3 ± 2.6	25 ± 3	35.4 (23)	23.7 ± 3.8
Normal (*n* = 373)	31.5 (28.7, 34.2)	5.0 ± 3.9	38.2 ± 2.9	7.8 ± 2.7	17 ± 4	16.9 (63)	22.9 ± 3.8
Overcoverage (*n* = 20)	43.0 (41.9, 44.1)	−4.4 ± 4.5	34.8 ± 2.9	5.0 ± 2.8	7 ± 3	10.0 (2)	20.8 ± 4.1
Whole Chort (*n* = 493)	30.2 (26.1, 33.9)	6.1 (2.9, 10.0)	38.9 ± 3.4	8.1 ± 2.8	18 (14, 22)	20.5 (101)	23.0 ± 3.8

*Note*: Continuous variable data presented as mean ± SD or median (Q1, Q3) Categorical variables data presented as % (*n*).

Abbreviations: BDDH = borderline developmental dysplasia of the hip; DDH = developmental dysplasia of the hip; FHEI = femoral head extrusion index; LCEA = lateral center‐edge angle.

**FIGURE 4:**
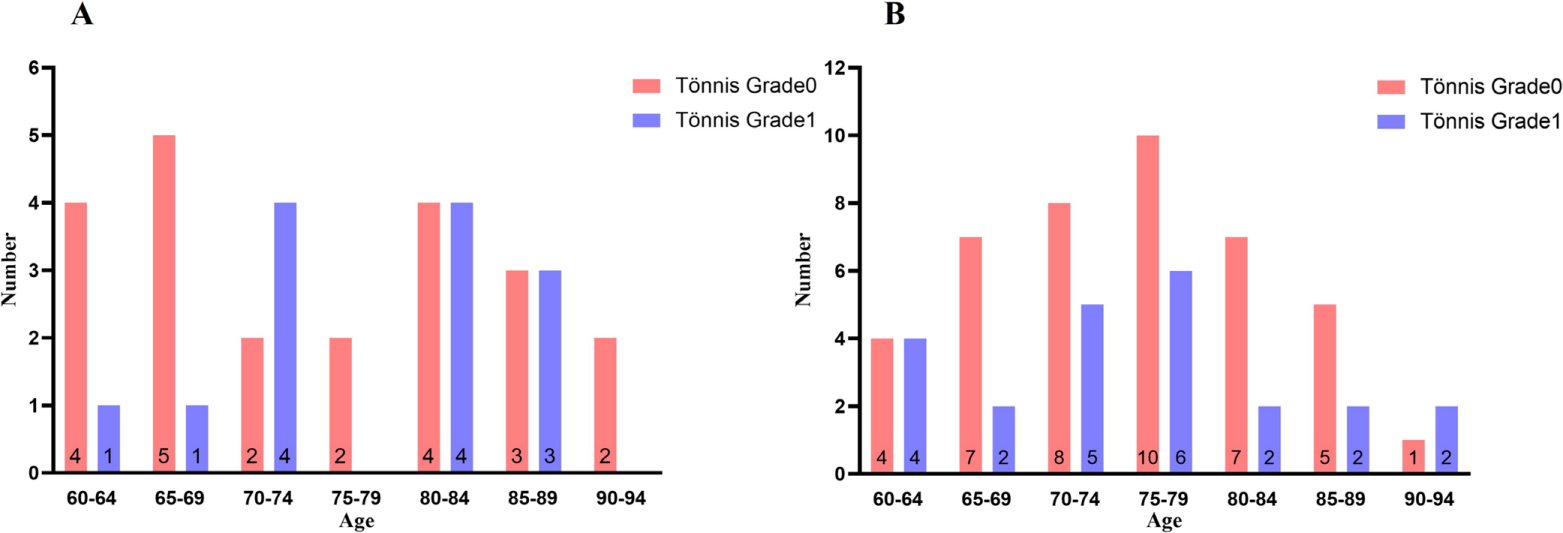
Number of patients with Tönnis Grade 0 and Tönnis Grade 1 in each age group in the DDH and BDDH groups. (A) Number of patients in the DHH group with Tönnis Grade 0 and Tönnis Grade 1 by age group. (B) Number of patients in the BDDH group with Tönnis Grade 0 and Tönnis Grade 1 by age group.

Imaging parameters were compared among the DDH, BDDH, and healthy groups (Table 2). Significant differences were observed in the Tönnis angle, Sharp's angle, femoral head lateralization, and FHEI between the normal group and both the DDH and BDDH groups. Further comparisons between the DDH and BDDH groups indicated no significant difference in femoral head lateralization and FHEI. Contrarily, the Tönnis angle and Sharp's angle were significantly larger in the DDH group than in the BDDH group. Additionally, 35.4% of patients in the DDH group had mild OA (Tönnis Grade 1), compared with 37.1% of patients in the BDDH group, both higher than the 16.9% observed in the normal group. Patients with DDH (odds ratio, OR = 2.42, 95% confidence interval, CI = 1.17–4.99) and BDDH (OR = 2.39, 95% CI = 1.36–4.21) were at a higher risk of developing mild OA, compared with normal individuals (OR = 0.36, 95% CI = 0.22–0.59). To further explore the association between the development of mild OA, DDH, and aging, patients were grouped into 5‐year age cohorts. The distribution pattern of Tönnis Grade 0 and 1 cases was analyzed (Figure 4). Patients with Tönnis Grade 0 DDH and BDDH were observed in all age groups, including two patients with DDH aged over 90 years. To identify the predictors of mild OA, including age, sex, LCEA, and Tönnis angle, multifactorial regression analysis was conducted (Table 3). Only the Tönnis angle was associated with mild OA (*β* = 0.076, *p* = 0.048, OR = 1.078, 95% CI: 1.001–1.162), with a 7.8% increase in OA risk for each 1° increase in the Tönnis angle. Considering that even mild OA might slightly alter measured angles (osteophytes, etc.). We analyzed only Tönnis grade 0 patients (*n* = 393) and found that the incidence of DDH (*n* = 23) was still as high as 5.6%, while that of BDDH (*n* = 42) was 10.7%.

**TABLE 2:** Comparison of imaging measurements between patients in the DDH group, patients in the BDDH group, and patients in the normal group.

Variable	Normal group (*n* = 373)	BDDH group (*n* = 65)	DDH group (*n* = 35)	*p*
Tönnis angle in °	5.0 ± 3.9	11.5 ± 4.1^a^	17.5 ± 4.1^a^, ^b^	< 0.001
Sharp's angle in °	38.2 ± 2.9	41.7 ± 2.5^a^	44.0 ± 2.5^a^, ^b^	< 0.001
Femoral head lateralization in mm	7.8 ± 2.7	9.3 ± 2.6^a^	10.3 ± 2.8^a^	< 0.001
FHEI in %	17 ± 4	25 ± 3^a^	30 ± 4^a^	< 0.001
Tönnis Grade 1	16.9 (63)	35.4 (23)^a^	37.1 (13)	< 0.001

*Note*: Continuous variable data presented as mean ± SD or median (Q1, Q3) Categorical variables data presented as % (*n*). For parameter comparisons between multiple groups, use ANOVA with post hoc analysis.

Abbreviations: BDDH = borderline developmental dysplasia of the hip; DDH = developmental dysplasia of the hip; FHEI = femoral head extrusion index; LCEA = lateral center‐edge angle.

^a^ Comparison with the normal group, *p* < 0.05.

^b^ Comparison with the BDDH group, *p* < 0.05.

**TABLE 3:** Results of multivariate logistic regression analysis.

Variable	*β*	*p*	OR (95% CI)
Age	0.016	0.249	1.016 (0.989, 1.044)
Sex (female)	−0.538	0.087	0.584 (0.315, 1.081)
LCEA	−0.011	0.811	0.989 (0.903, 1.083)
Tönnis angle	0.076	0.048	1.078 (1.001, 1.162)
Sharp's angle	−0.030	0.576	0.971 (0.874, 1.078)
Femoral head lateralization	0.027	0.590	1.027 (0.932, 1.132)
FHEI	1.289	0.709	3.631 (0.004, 3180.48)

Abbreviations: BDDH = borderline developmental dysplasia of the hip; DDH = developmental dysplasia of the hip; FHEI = femoral head extrusion index; LCEA = lateral center‐edge angle.

Finally, BMI did not differ among the DDH, BDDH, and normal groups (22.9 ± 3.9 vs. 23.7 ± 3.8 vs. 22.9 ± 3.8, *p* = 0.36).

Furthermore, the distribution of parameters other than LCEA was analyzed in the DDH and normal groups. Acetabular anatomy was assessed using Sharp's angle and the Tönnis angle, whereas the position of the femoral head relative to the acetabular floor was assessed using femoral head lateralization and FHEI. A Tönnis angle > 14°, Sharp's angle > 43°, femoral head lateralization > 10 mm, and FHEI > 27% were the additional DDH diagnostic criteria [21, 22]. The number and proportion of patients with DDH, BDDH, and normal hips exhibiting abnormalities in each of these parameters were calculated (Table 4). The proportion of patients with abnormalities across all parameters was significantly higher in the DDH and BDDH groups than in the normal group. Additionally, the proportion of abnormalities in the Tönnis angle, Sharp's angle, and FHEI was significantly higher in the DDH group than in the BDDH group.

**TABLE 4:** Abnormalities of indicators in patients in the DDH group, patients in the BDDH group and patients in the normal group.

Variable	Normal group (*n* = 373)	BDDH group (*n* = 65)	DDH group (*n* = 35)	*p*
Tönnis angle	0.3 (1)	21.5 (14)^a^	71.4 (28)^a^, ^b^	< 0.001
Sharp's angle	4.0 (15)	27.6 (18)^a^	62.9 (19)^a^, ^b^	< 0.001
Femoral head lateralization	21.7 (81)	36.9 (24)^a^	48.6 (17)^a^	< 0.001
FHEI	0.2 (1)	26.2 (17)^a^	74.2 (26)^a^, ^b^	< 0.001

*Note*: Categorical variables data presented as % (*n*). For parameter comparisons between multiple groups, use ANOVA with post hoc analysis.

Abbreviations: BDDH = borderline developmental dysplasia of the hip; DDH = developmental dysplasia of the hip; FHEI = femoral head extrusion index; LCEA = lateral center‐edge angle.

^a^ Comparison with the normal group, *p* < 0.05.

^b^ Comparison with the BDDH group, *p* < 0.05.

In the BDDH group, 14 patients (21.5%) had a Tönnis angle > 14°, 18 (27.6%) had a Sharp's angle > 43°, and 6 (9.2%) had both abnormalities. Moreover, 24 patients (36.9%) had femoral head lateralization > 10 mm, 17 (26.2%) had an FHEI > 27%, and 7 (10.7%) had both abnormalities. In the DDH group, 31 patients (88.5%) had a Tönnis angle > 14°, 22 (62.9%) had a Sharp's angle > 43°, and 19 (54.2%) had both abnormalities. Additionally, 17 (48.5%) patients had femoral head lateralization > 10 mm, 26 (74.2%) had an FHEI > 27%, and 14 (40%) had both abnormalities.

In the BDDH group, 17 patients (26.1%) had all four parameters within the normal range, 27 (41.5%) had one abnormal parameter, 18 (27.7%) had two abnormal parameters, and 3 (2.9%) had three abnormal parameters. No patient had abnormalities in all four parameters. In the DDH group, 1 (2.9%) patient had all four parameters within the normal range, 2 (5.7%) had one abnormal parameter, 14 (40%) had two abnormal parameters, and 9 each (25.7%) had three and four abnormal parameters.

Both intra‐ and inter‐observer agreements were excellent (Table 5).

**TABLE 5:** Intra‐ and inter‐observer consistency tests.

	LCEA	Tönnis angle	Sharp's angle	Femoral head lateralization	FHEI
Intra‐observer	0.96 (0.95–0.97)	0.98 (0.97–0.98)	0.93 (0.92–0.94)	0.93 (0.92–0.94)	0.97 (0.96–0.97)
Inter‐observer	0.91 (0.90–0.93)	0.95 (0.94–0.96)	0.93 (0.92–0.94)	0.94 (0.93–0.95)	0.92 (0.90–0.93)

Abbreviations: FHEI = femoral head extrusion index; LCEA = lateral center‐edge angle.

## 4 Discussion

### 4.1 The Prevalence of DDH and BDDH Among Asymptomatic Adults

In our study, 7.1% of individuals aged > 60 years with unilateral femoral neck fractures had imaging results consistent with DDH, whereas 13.2% had results consistent with BDDH.

### 4.2 The Relationship Between DDH and BDDH With Mild OA

Individuals with DDH or BDDH were more likely to exhibit mild OA symptoms. However, not all individuals with DDH or BDDH develop OA. A higher Tönnis angle—but not decreased LCEA—increased the risk of developing OA.

### 4.3 Hip Morphology in Individuals With DDH or BDDH

This study further explored hip morphology in individuals with DDH and the mechanism by which functional hips are maintained without severe OA. The LCEA reflects only lateral coverage; therefore, the acetabular structure was assessed using the Tönnis angle and Sharp's angle. Moreover, the position of the femoral head relative to the acetabulum was evaluated using femoral head lateralization and the FHEI. These four imaging parameters significantly differed between the DDH and BDDH groups, compared with the normal group. Interestingly, the Tönnis angle and Sharp's angle—which evaluate the acetabular structure—significantly differed between the DDH and BDDH groups, indicating their function in characterizing hip morphology.

The proportion of abnormal parameters significantly differed between the DDH and BDDH groups and the normal group upon using the Tönnis angle as an additional diagnostic parameter. Additionally, the proportion of patients with an abnormal Tönnis angle, Sharp's angle, and FHEI was significantly higher in the DDH group than in the BDDH group. There was no significant difference in femoral head lateralization between the DDH and BDDH groups, likely because we used 10 mm as the upper limit of normal based on European and American literature, a criterion that may not be applicable to the Chinese population. The femoral head lateralization in our subjects was generally small, with a mean value of 8.1 mm. This is a limitation of our study. To summarize, over 60% of patients in the BDDH group had no more than one abnormal parameter, whereas over 90% in the DDH group had at least two abnormal parameters. A combined assessment using multiple imaging parameters may facilitate better characterization of hip morphology in DDH and BDDH.

### 4.4 Clinical Significance

The natural history of DDH in asymptomatic populations remains unknown. If the risk of OA in a hip meeting the imaging criteria for DDH is high, prophylactic intervention may be necessary even in asymptomatic patients [23]. In contrast, if individuals with imaging features of DDH maintain healthy hip function into old age without progressing to severe OA, they do not require advanced therapy. This paper provides guidance for clinicians in the screening and management of patients with DDH.

### 4.5 Strengths and Limitations

In this study, patients aged above 60 years with unilateral femoral neck fractures were selected. In China, 60 years is the legal retirement age; therefore, older adults participate less frequently in high‐intensity work or exercise. Thus, the probability of developing OA secondary to hip hypermobility decreases. In this study, anteroposterior pelvic radiographs of older adults with unilateral femoral neck fractures were utilized to assess contralateral hip morphology. This technique facilitated analyzing a large sample size for valid assessment. The large sample size of this study facilitates a more precise estimation of the prevalence rates of DDH and BDDH.

This study has some limitations. First, we must acknowledge that using patients with unilateral femoral neck fractures as the study population may introduce selection bias: these individuals may not perfectly represent the general elderly population. For example, compared to the general population, patients with femoral neck fractures tend to have a higher proportion of female patients, lower bone density, poorer nutritional status, and may have anatomical abnormalities of the hip joint [24]. However, there is currently no direct evidence to suggest that DDH deformity increases or decreases the risk of femoral neck fractures and our study was not designed to assess any relationship between hip dysplasia and fracture occurrence. Although female patients predominantly require surgical intervention for DDH, this study found no significant difference in the incidence of DDH and BBDH among asymptomatic male and female participants (7.6% vs. 6.9%, *p* = 0.80; 13.6% vs. 13.0%, *p* = 0.86). This further demonstrates that DDH is not exclusively a female‐specific issue. Therefore, we believe it is reasonable to use patients with unilateral femoral neck fractures to estimate the incidence of DDH and BBDH in the general elderly population. Second, it included only patients with unilateral femoral neck fractures. Because femoral neck fractures and hemorrhages may lead to displacement and deformation of the femoral head, only the fractured contralateral hip was assessed. This makes it impossible to determine the proportion of bilateral hip dysplasia. Additionally, dysplasia affecting the fractured side might have been overlooked. Therefore, we acknowledge that we may have underestimated the incidence of DDH/BDDH. Third, only imaging data were analyzed without considering patients' occupations or exercise habits. Low levels of physical activity in some patients may prevent dysplasia from progressing to OA, which could have affected our results. Additionally, only anteroposterior pelvic radiographs—which can only capture lateral coverage—were analyzed. We did not measure data such as the anterior coverage of the acetabulum and the femoral anteversion angle in patients, which also influence hip joint stability. This may explain why the significant abnormalities in parameters such as FHEI observed in the DDH group did not result in severe OA, as the hip joint gained sufficient coverage in other directions to ensure its stability. Finally, clinical symptoms and physical examination results to assess the hip joint were not documented. This limits our ability to identify factors that prevent progression to severe osteoarthritis in abnormal hip joints. Further analysis of factors such as the patient's genetic makeup, activity level, or cartilage characteristics is needed to refine our research.

### 4.6 Research on the Incidence of DDH and BDDH

Several studies have discussed the prevalence of DDH in the general population (Table 6). Jacobsen et al. [1] conducted a prospective longitudinal cohort study in Copenhagen, including 3859 asymptomatic individuals (7718 hips) aged 22–93 years. They reported that 19.2% of hips were categorized as BDDH and 3.4% as DDH. Engesæter et al. [12] conducted a prospective cohort study, including 2072 asymptomatic 19‐year‐old Norwegians (4144 hips). They diagnosed 16.7% of participants with BDDH and 3.3% with DDH. Leide et al. [14] evaluated anteroposterior pelvic radiographs from 1870 residents aged 20–70 years in Malmö, Sweden; they reported a prevalence of 5.2% for DDH and 16.2% for BDDH, without differences between men and women. Raveendran et al. [15] reported data from the Johnston County Osteoarthritis Program in the United States, including 1601 asymptomatic individuals (3202 hips). DDH was diagnosed in 7.6% of hips and 9.4% of individuals, whereas BDDH was diagnosed in 18.8% of hips and 25.1% of individuals. Kim et al. [13] retrospectively analyzed prospectively collected data from 200 (400 hips) asymptomatic volunteers aged 18 to 50 years in South Korea. They reported a 15% prevalence of DDH. In older adults, Andersen et al. [23] demonstrated that approximately 3% of older athletes with good hip function had an LCEA < 20°. Cheng et al. [25] studied 427 Japanese community residents (279 women and 148 men) aged 50–96 years who had undergone health screenings. Of them, 21.3% had an LCEA < 25°. A 2024 meta‐analysis of 14 studies analyzed 10,998 hips from 5506 asymptomatic individuals; DDH had an overall prevalence of 2.3% [26]. Nonetheless, the prevalence of DDH in the Chinese general population remains unknown. In the present study, 20.3% of individuals aged > 60 years with unilateral femoral neck fractures had an LCEA < 25°.

**TABLE 6:** Studies of the prevalence of DDH and BDDH in the general population.

Lead author (year)	Location	Population	Age, years	Patients (hips), *n*	Sex	DDH	BDDH
Jacobsen (2005) [1]	Denmark	Population‐based cohort; no OA exclusion	22–93	3859 (7718)	63% female	3.4% by hip	19.2% by hip
Engesæter (2013) [12]	Norway	Invited follow‐up of population‐based cohort; no OA exclusion	19	2072 (4144)	56% female	3.3% by patient	16.7% by hip
Raveendran (2018) [15]	USA	Population‐based cohort; OA exclusion	> 45	1601 (3202)	57% female	7.6% by hip 9.4% by patient	18.8% by hip 25.1% by patient
Kim (2019) [13]	Korea	Invited follow‐up of population‐based cohort; OA exclusion	18–50	200 (400)	64% female	15% by patient	—
Leide (2021) [14]	Sweden	Population‐based cohort; no OA exclusion	20–70	1870 (3740)	63% female	5.2% by patient	16.2% by patient
Cheng (2022) [25]	Japan	Invited follow‐up of population‐based cohort; no OA exclusion	50–96	427 (854)	65% female	21.3% by patient	

Abbreviations: BDDH = borderline developmental dysplasia of the hip; DDH = developmental dysplasia of the hip.

### 4.7 Conclusions

In conclusion, the prevalence of DDH imaging abnormalities is not low among Chinese older adults with no OA development. Therefore, we do not recommend DDH screening for asymptomatic individuals or early intervention surgery. Individuals with DDH and BDDH are more likely to present with mild OA symptoms, but not all individuals develop OA. Using multiple imaging parameters along with LCEA facilitates the characterization of hip morphology in asymptomatic individuals with DDH.

## Author Contributions

We thank all authors who contributed to this work. All authors have made substantial contributions to (1) the study conception and design (2) drafting and revising critically for important intellectual content, and (3) final approval of the submitted version. D.L, W.C. and H.Z. were primarily responsible for the supervision of the research, including research protocol designing, data acquisition, and manuscript preparation Z.C. and Z.Z. were mainly responsible for research design, data extraction, statistical analysis, article analysis, and manuscript drafting. Z.C. and Z.Z. contributed to this work equally and both of them were co‐first authors. H.C. and Z.X. participated in this study design, manuscript drafting, and data analysis.

## Ethics Statement

The study was approved by our hospital ethics review board (number 2023KY002‐KS001). The study was performed at the senior department of Orthopedics, the fourth medical center of the Chinese PLA General Hospital. We received the ethics committee review board approval from our institution before the initiation of this study.

## Conflicts of Interest

The authors declare no conflicts of interest.

## Data Availability

The data that support the findings of this study are available from the corresponding author upon reasonable request.

## References

[1] S. Jacobsen , S. Sonne‐Holm , K. Søballe , P. Gebuhr , and B. Lund , “Hip Dysplasia and Osteoarthrosis: A Survey of 4151 Subjects From the Osteoarthrosis Substudy of the Copenhagen City Heart Study,” Acta Orthopaedica 76, no. 2 (2005): 149–158. 16097538 10.1080/00016470510030517

[2] S. B. Murphy , R. Ganz , and M. E. Müller , “The Prognosis in Untreated Dysplasia of the Hip. A Study of Radiographic Factors That Predict the Outcome,” Journal of Bone and Joint Surgery. American Volume 77, no. 7 (1995): 985–989. 7608241 10.2106/00004623-199507000-00002

[3] C. C. Wyles , M. J. Heidenreich , J. Jeng , D. R. Larson , R. T. Trousdale , and R. J. Sierra , “The John Charnley Award: Redefining the Natural History of Osteoarthritis in Patients With Hip Dysplasia and Impingement,” Clinical Orthopaedics and Related Research 475, no. 2 (2017): 336–350. 27071391 10.1007/s11999-016-4815-2 PMC5213917

[4] M. B. Millis and Y. J. Kim , “Rationale of Osteotomy and Related Procedures for Hip Preservation: A Review,” Clinical Orthopaedics and Related Research 405 (2002): 108–121. 10.1097/00003086-200212000-00013 12461362

[5] N. Fredensborg , “The CE Angle of Normal Hips,” Acta Orthopaedica Scandinavica 47, no. 4 (1976): 403–405. 961394 10.3109/17453677608988709

[6] S. Jacobsen and S. Sonne‐Holm , “Hip Dysplasia: A Significant Risk Factor for the Development of Hip Osteoarthritis. A Cross‐Sectional Survey,” Rheumatology (Oxford, England) 44, no. 2 (2005): 211–218. 15479751 10.1093/rheumtology/keh436

[7] G. Wiberg , “Studies on Dysplastic Acetabula and Congenital Subluxation of the Hip Joint With Special Reference to the Complication of Osteo‐Arthritis,” Journal of the American Medical Association 115, no. 1 (1940): 81.

[8] J. Lara , A. Neira , A. Garín , et al., “Anatomical Insights Beyond the Centre Edge Angle in Borderline Hip Dysplasia: A Computerised Tomography Study,” Journal of Experimental Orthopaedics 12, no. 2 (2025): e70268. 40390852 10.1002/jeo2.70268 PMC12086787

[9] N. Ramadanov , M. Voss , R. Hable , et al., “Periacetabular Osteotomy Versus Hip Arthroscopy in Patients With Borderline Developmental Dysplasia of the Hip: A Systematic Review and Multi‐Level Meta‐Analysis,” Journal of Experimental Orthopaedics 12, no. 3 (2025): e70311. 40612035 10.1002/jeo2.70311 PMC12221243

[10] J. Zhang , C. Li , J. Zhang , G. Zhao , and Y. Liu , “Lateral Center‐Edge Angle of 18° (Bone‐Edge): Threshold for Hip Arthroscopy Treatment in Patients With Borderline Developmental Dysplasia of the Hip?,” Orthopaedic Surgery 15, no. 10 (2023): 2665–2673. 37641583 10.1111/os.13877 PMC10549843

[11] B. D. Kuhns , N. Becker , M. J. Strok , E. J. O'Brien , M. Hassan , and B. G. Domb , “Patient‐Reported Outcomes After Periacetabular Osteotomy Versus Hip Arthroscopy for Borderline Acetabular Dysplasia Are Both Favorable: A Systematic Review,” Arthroscopy 41, no. 8 (2025): 3079–3093.e2. 39672243 10.1016/j.arthro.2024.11.090

[12] I. Engesæter , L. B. Laborie , T. G. Lehmann , et al., “Prevalence of Radiographic Findings Associated With Hip Dysplasia in a Population‐Based Cohort of 2081 19‐Year‐Old Norwegians,” Bone & Joint Journal 95‐b, no. 2 (2013): 279–285. 10.1302/0301-620X.95B2.30744 23365042

[13] C. H. Kim , J. I. Park , D. J. Shin , S. H. Oh , M. Y. Jeong , and P. W. Yoon , “Prevalence of Radiologic Acetabular Dysplasia in Asymptomatic Asian Volunteers,” Journal of Hip Preservation Surgery 6, no. 1 (2019): 55–59. 31069096 10.1093/jhps/hnz001 PMC6501437

[14] R. Leide , A. Bohman , D. Wenger , S. Overgaard , C. J. Tiderius , and C. Rogmark , “Hip Dysplasia Is Not Uncommon but Frequently Overlooked: A Cross‐Sectional Study Based on Radiographic Examination of 1,870 Adults,” Acta Orthopaedica 92, no. 5 (2021): 575–580. 34238106 10.1080/17453674.2021.1936918 PMC8519544

[15] R. Raveendran , J. L. Stiller , C. Alvarez , et al., “Population‐Based Prevalence of Multiple Radiographically‐Defined Hip Morphologies: the Johnston County Osteoarthritis Project,” Osteoarthritis and Cartilage 26, no. 1 (2018): 54–61. 29024801 10.1016/j.joca.2017.10.002 PMC5732866

[16] S. M. Freiman , M. T. Schwabe , L. Fowler , J. C. Clohisy , and J. J. Nepple , “Prevalence of Borderline Acetabular Dysplasia in Symptomatic and Asymptomatic Populations: A Systematic Review and Meta‐Analysis,” Orthopaedic Journal of Sports Medicine 10, no. 2 (2022): 23259671211040455. 35155698 10.1177/23259671211040455 PMC8832597

[17] S. Jacobsen , S. Sonne‐Holm , B. Lund , et al., “Pelvic Orientation and Assessment of Hip Dysplasia in Adults,” Acta Orthopaedica Scandinavica 75, no. 6 (2004): 721–729. 15762262 10.1080/00016470410004094

[18] D. Tönnis , “Normal Values of the Hip Joint for the Evaluation of X‐Rays in Children and Adults,” Clinical Orthopaedics and Related Research, no. 119 (1976): 39–47. 954321

[19] I. Sharp , “Acetabular Dysplasia: The Acetabular Angle,” Journal of Bone and Joint Surgery. British Volume 43 (1961): 268–272.

[20] C. H. Heyman and C. H. Herndon , “Legg‐Perthes Disease; a Method for the Measurement of the Roentgenographic Result,” Journal of Bone and Joint Surgery, American Volume 32 a, no. 4 (1950): 767–778. 14784485

[21] J. C. Clohisy , J. C. Carlisle , P. E. Beaulé , et al., “A Systematic Approach to the Plain Radiographic Evaluation of the Young Adult Hip,” Journal of Bone and Joint Surgery. American Volume 90, no. Suppl 4 (2008): 47–66. 10.2106/JBJS.H.00756 PMC2682767 18984718

[22] M. Tannast , M. S. Hanke , G. Zheng , S. D. Steppacher , and K. A. Siebenrock , “What Are the Radiographic Reference Values for Acetabular Under‐ and Overcoverage?,” Clinical Orthopaedics and Related Research 473, no. 4 (2015): 1234–1246. 25384429 10.1007/s11999-014-4038-3 PMC4353515

[23] L. A. Anderson , M. B. Anderson , A. Kapron , et al., “The 2015 Frank Stinchfield Award: Radiographic Abnormalities Common in Senior Athletes With Well‐Functioning Hips but Not Associated With Osteoarthritis,” Clinical Orthopaedics and Related Research 474, no. 2 (2016): 342–352. 26054483 10.1007/s11999-015-4379-6 PMC4709310

[24] T. Sasagawa , N. Yokogawa , H. Hayashi , et al., “A Multicenter Study of 1‐Year Mortality and Walking Capacity After Spinal Fusion Surgery for Cervical Fracture in Elderly Patients,” BMC Musculoskeletal Disorders 23, no. 1 (2022): 798. 35987644 10.1186/s12891-022-05752-5 PMC9392237

[25] V. K. Cheng , M. Hasegawa , T. Hattori , et al., “Prevalence of Radiographic Hip Dysplasia in Japanese Population‐Based Study,” Modern Rheumatology 32, no. 2 (2022): 438–443. 33910453 10.1080/14397595.2021.1918884

[26] K. P. O'Connor , B. J. Marshall , J. Davison , J. C. Clohisy , and M. C. Willey , “Prevalence of Radiographic Hip Dysplasia in the General Adult Population: A Systematic Review,” Iowa Orthopaedic Journal 44, no. 1 (2024): 145–149. PMC11195875 38919354

